# Prognostics of multiple malaria episodes and nutritional status in children aged 6 to 59 months from 2013 to 2017 in Dangassa, Koulikoro region, Mali

**DOI:** 10.1186/s12936-024-04999-8

**Published:** 2024-06-13

**Authors:** Soumba Keita, Oumar Thiero, Mahamoudou Toure, Fousseyni Kane, Moussa Keita, Ibrahim Sanogo, Drissa Konate, Daouda Sanogo, Sory Ibrahim Diawara, Hamady Coulibaly, Sidibé M.’Baye Thiam, Nafomon Sogoba, Mahamadou Diakite, Seydou Doumbia

**Affiliations:** 1 West African International Center for Excellence in Malaria Research, University of Sciences, Techniques and Technologies of Bamako, Bamako, Mali; 2 University Clinical Research Center (UCRC), University of Sciences, Techniques and Technologies of Bamako, UCRC-USTTB / Point-G, 1805 Bamako, Mali; 3 Malaria Research and Training Center (MRTC), University of Sciences, Techniques and Technologies of Bamako, Bamako, Mali; 4 Department of Health Research and Education, Faculty of Medicine and Odonto Stomatology, University of Sciences, Techniques and Technologies of Bamako (USTTB), Bamako, Mali

**Keywords:** Malaria, Anemia, Underweight, Nutritional status, Multiple episodes

## Abstract

**Background:**

In Africa, the relationship between childhood nutritional status and malaria remains complex and difficult to interpret. Understanding it is important in the improvement of malaria control strategies. This study aimed to assess the influence of nutritional status on the occurrence of multiple malaria episodes in children aged 6 to 59 months between 2013 and 2017 living in the village of Dangassa, Mali.

**Methods:**

A community-based longitudinal study was conducted using cross-sectional surveys (CSSs) at the beginning (June) and end (November) of the malaria transmission season associated with passive case detection (PCD) at the Dangassa Community Health Centre. Children with asymptomatic malaria infection during cross-sectional surveys were selected and their malaria episodes followed by PCD. Malaria indicators in person-months were estimated using an ordinal-logistic model repeated on subjects during follow-up periods.

**Results:**

The incidence rate (IR) during the period of high transmission (June to October), for 1 episode and for 2 + episodes peaked in 2013 with 65 children (IR = 95.73 per 1000 person-months) and 24 cases (IR = 35.35 per 1000 person-months), respectively. As expected, the risk of multiple episodes occurring during the period of high transmission was 3.23 compared to the period of low transmission after adjusting for other model parameters (95% CI [2.45–4.26], p = 0.000). Children with anaemia were at high risk of having multiple episodes (OR = 1.6, 95% CI [1.12–2.30], p = 0.011). However, the risk of having 2 + episodes for anemic children was higher during the period of low transmission (RR = 1.67, 95% CI [1.15–2.42], p = 0.007) compared to the period of high transmission (RR = 1.58, 95% CI [1.09–2.29], p = 0.016). The trend indicated that anemic and underweight children were significantly associated with multiple malaria episodes during the period of low transmission (p < 0.001).

**Conclusion:**

Results show that multiple episodes of malaria are significantly related to the nutritional status (anaemia and underweight) of the child during the two transmission seasons and more pronounced during the dry season (period of low transmission). Further research including other malnutrition parameters will be needed to confirm these findings.

## Background

Malaria, anaemia, and malnutrition are responsible for more than 50% of child mortality in the world, particularly among children under 5 years of age [1–3]. Malaria remains the leading cause of morbidity and mortality in Malian children under 5 years of age, with an overall prevalence of 19%, according to the malaria indicator survey (MIS) of 2021 [4]. The Koulikoro district is one of the third with a prevalence above the country mean prevalence (23%), following Mopti (27%), and Sikasso (26%) [4].

In Mali, besides malaria, malnutrition remains a major cause of morbidity and mortality among children under 5 years of age. Micronutrient deficiencies, known as the main cause of malnutrition, is highly prevalent during the malaria transmission season which last from June to October each year [5]. Most of the death from undernourished children, are due to common infections associated with immunodeficiency [6]. In Mali, it affects 27% of children under 5 years of age in chronic form, 19% of whom are underweight (“low weight for age”) [7]. Being a composite indicator, underweight reflects both stunting and wasting, with no differentiation between them. Therefore, children presenting a low weight for their age are both growths stunted and wasted [8].

As one of one of the most common public health problems in the world, and especially one of the complications of malaria infection in endemic regions, anaemia plays a major role in malaria morbidity and mortality [9, 10]. It is multifactorial, affecting 40% of children under 5 years of age, and half of these cases are attributed to nutritional iron deficiency [11]. More than 82% of children under 5 years in Mali suffer from anaemia, with 25% suffering from mild anaemia, 51% from moderate, and 6% from severe anaemia [7].

Children’s nutritional status is a sensitive indicator of community health. The effects of malnutrition in children are long-lasting and extend decades beyond childhood [8]. However, the relationship between nutritional status and clinical malaria, on the one hand, and anaemia on the other, remains complex and difficult to interpret. Anaemia has not been found associated with the frequency of malaria episodes, but it has been demonstrated to be significantly associated with malnutrition [12]. In contrast, other studies have shown that malnutrition and anaemia are associated with a higher risk of *Plasmodium* infection and infectious episodes [13, 14].

The relationship between nutritional status, anaemia, and malaria episodes among children under the age of five is not well documented in Mali. This study aimed to investigate longitudinally the influence of nutritional status on multiple episodes of clinical malaria in children aged 6–59 months asymptomatic to malaria in Dangassa, health district of Kati, from 2013 to 2017.

## Methods

### Study area

**Fig. 1:**
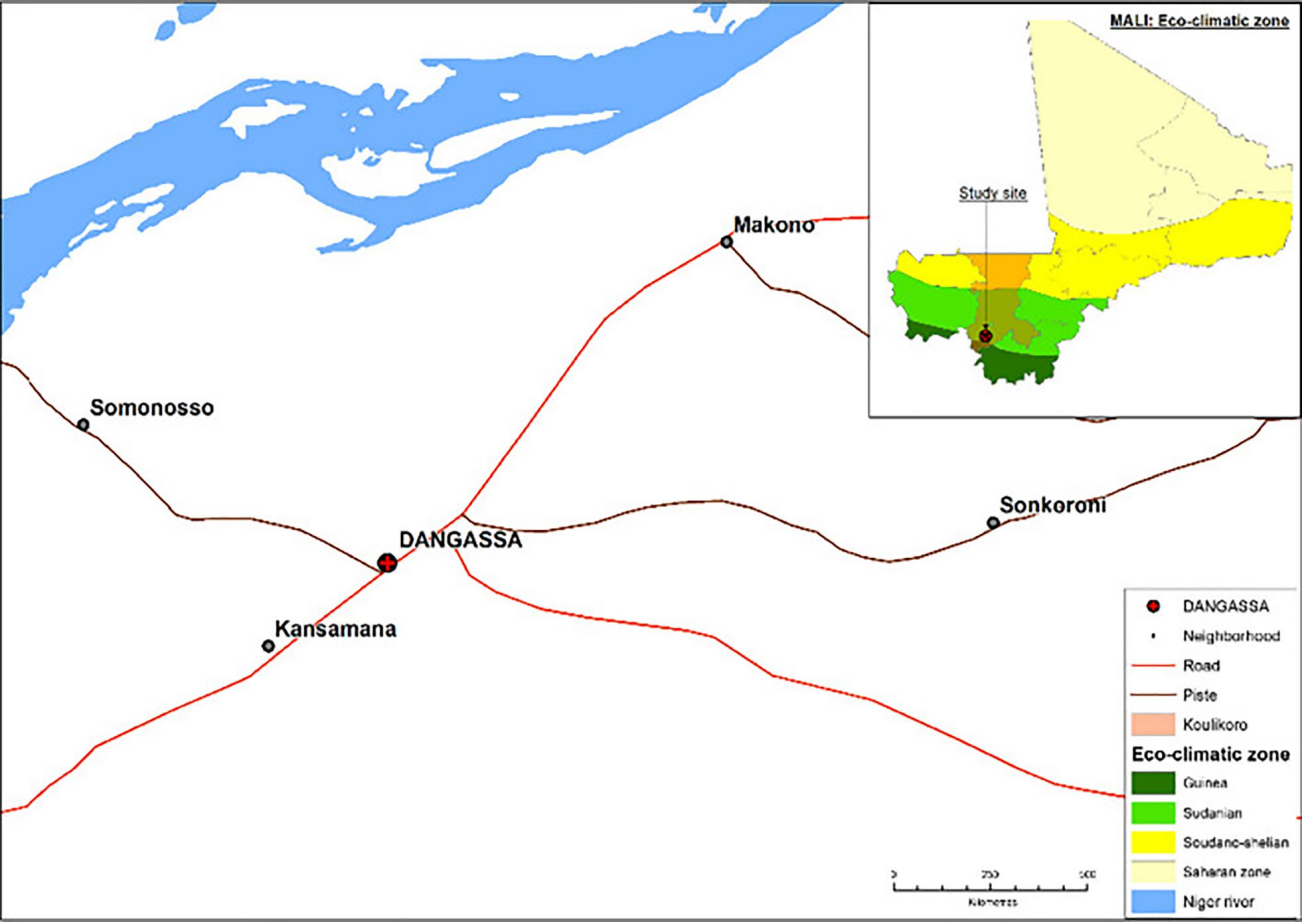
Study site of Dangassa, Oueléssébougou health district, Koulikoro region

The study was conducted in Dangassa (8° 12' 37.253'' W and 12° 8' 46.279' N) which is located 75 km southwest of Bamako in the health district of Ouéléssebougou, Koulikoro region (Fig. 1). This is a Sudan savannah eco-geographical zone with a rainy season lasting from June to October and a dry season from November to May. The dominant winds are the monsoon (rainy season) and the harmattan (dry season). Malaria transmission is seasonal in Dangassa, with 6 to 7 months of transmission per year (June to December). In Dangassa at the start of malaria transmission season (June-July), the prevalence of *Plasmodium falciparum* for children under 5 years of age was respectively 59.4%, 32.9% and 19.1% in 2014, 2015 and 2016 [15].

## Study design

### Cross-sectional surveys

Asymptomatic children were selected from the cross-sectional survey (CSSs) data. The selected children were used as a baseline for the follow-up of malaria cases at the community health center. Seven CSSs were performed: in June (at the start of the rainy season) and in October/November (at the end of the rainy season). However, in 2013 the only CSSs was performed in February, which was utilized as baseline for June and October for PCD. During each survey, demographic and clinical data were collected from each participant. Microscopy (smear) was used to assess the *P. falciparum* infection, while HemoCue^®^ Hb 301 was used to measure haemoglobin levels. The Weight was measured using a weigh scale. During the CSSs, only participants with fever (defined as axillary temperature ≥ 37.5 °C or fever within the previous 48 h) were tested for malaria using malaria rapid diagnostic test (RDT). Participants tested positive for malaria were provided treatment according to the policies of the Malian National Malaria Control Programme (NMCP).

### Passive case detection (PCD)

**Fig. 2:**
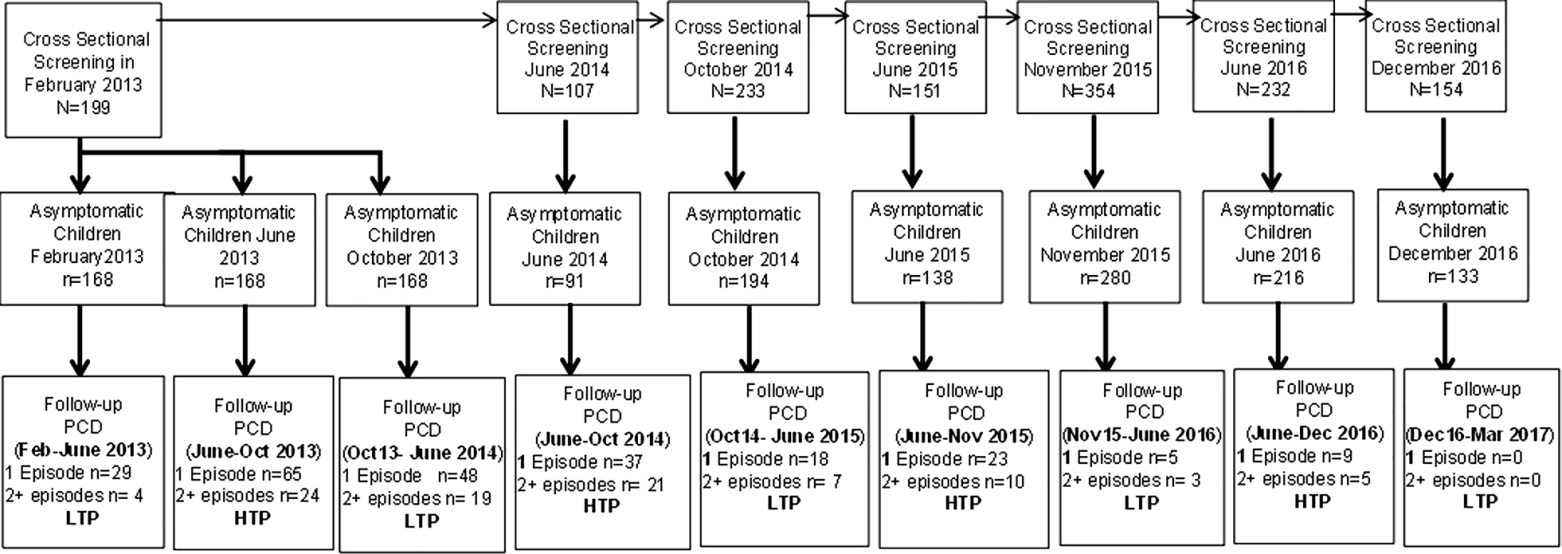
Follow up using Passive Case Detection (PCD) of children under 5 years for multiples malarial episodes during Low and High Transmission Periods (LTP and HTP) in Dangassa, from 2013 to 2017

Asymptomatic children identified during CSSs were followed up by passive case detection (PCD). Malaria incidence was determined after each CSSs (Fig. 2). From February 2013 to March 2017, a physician and a biologist followed cohort participants for malaria incidence through community health-based passive surveillance. Therefore, head and mothers of children selected for the study were informed to visit the health centre if they developed fever or other symptoms of malaria. Study participants with fever (suspected malaria cases) are tested using RDT and microscopy. All confirmed cases (RDT positive cases) were treated free of charge in accordance with national recommendations for malaria treatment. In addition, all severe cases were referred to the district health centre at the project's expense. During this follow-up, data on anaemia and malnutrition were collected in the same way as during the cross-sectional surveys.

### Operational definitions

- Malaria episode: Malaria episode for a participant was defined as new one only when time between two subsequent infections was greater than 20 days. The number of episodes per child was classified into three categories 0, 1 and 2 or more.
- Malnutrition indicator: ENA For Smart software was used to calculate scores (z-scores) based on the national results at the World Health Organization (WHO) reference population. Malnutrition indicators were determined using a standard deviation of −2 standard deviations or Z-score. The weight-for-age (WAZ) of children was classified in two categories, Z-score according to WHO growth standards references for children aged zero to 5 years 16. Score ≤ −2 standard deviation (SD) were defined as underweight, while score > −2 standard were defined as normal from the reference mean.
- Anaemia: anaemia was defined as a haemoglobin level < 11 g/dl (16). It was classified into two categories: anaemia: Hb level < 11 g/dl; no anaemia: Hb level ≥ 11 g/dl 18.

### Laboratory methods

Blood films were stained with 10% Giemsa and examined under a 100X oil immersion objective lens of a light microscope. Asexual parasites were estimated based on the number of parasitized red blood cells per 300 white blood cells multiplied by 25, assuming an average white blood cell count of 7500/µl. A minimum of 100 fields were examined before a thick drop was considered negative by two different experienced microscopists having read each smear separately. RDTs (*BIOLANE*^*®*^*)* for *P. falciparum* infection were performed according to the manufacturer's instructions. RDTs used were those currently recommended by the NMCP of Mali.

Haemoglobin concentration was measured in venous blood using HemoCue 301. Hemocue^®^ 301 is an easy to use device for field workers with a long-life span of the batteries which is important in rural areas where electricity is not always available.

### Statistical methods

Microsoft Access 2019 was used for database management and to track the malaria episode using PCD data between two CSSs. The data and graphical analysis were done using SAS version 9.2 and SPSS version 28. The person month incidence was adjusted for different time intervals: from June through October/November, from October/November through December and from December through June of the following year (2014–2016). In 2013, the times intervals were from February to June, then from June to October/November and from October/November to December. In 2017, the follow up time was from December 2016 to March 2017.

The yearly numbers of malaria episodes in person-months were adjusted using repeated ordinal-logistic regression model. The parameters were estimated using Generalized Estimating Equation (GEE) with the hybrid method (in which Fisher scoring iterations are performed before switching to the Newton–Raphson method). The maximum likelihood method was used for the algorithm convergence. The working correlation matrix has shown an exchangeable structure. The repeated subject effect was the children study ID with the intrasubject effect as the follow-up period. The p-value for analysis was set to 5%. The convergence criteria were satisfied, and the data fit well the model. The children exposure times within each transmission period were computed as the offset. The outcome variable for the model was the episodes numbers with three categories levels 0, 1 or 2 + episodes. Five risk factors: age groups, gender, anaemia status, weight for age (WAZ) and transmission period were used. From the model, the predict Incidence rate (IR), odd ratio (OR) and/or the relative risk (RR) for having higher malaria-episodes numbers were estimated.

## Results

Seven CSSs, from 2013 (February), 2014 (June, October), 2015 (June, November) and 2016 (June, December), were carry-out during which 199,107,233,151,324,232 and 154 children were respectively enrolled at the beginning of each of the malaria-transmission-periods. The prevalence of clinical malaria was 15.6% in 2013, 15.0% vs 16.7%, 8.6% vs 13.6%, and 6.9% vs 13.6%, respectively at the start and end of transmission in 2014, 2015, and 2016 (Table 1).

**Table 1:** Demographics and clinicals characteristics at the cross-sectional survey for children under 5 years from 2013 to 2016 in Dangassa

Age groupsin months	Feb 2013 n (%)	Jun 2014 n (%)	Oct 2014 n (%)	June 2015 n (%)	Nov 2015 n (%)	Jun 2016 n (%)	Dec 2016 n (%)
	N = 199	N = 107	N = 233	N = 151	N = 324	N = 232	N = 154
Less than 36	47 (23.6)	1 (0.9)	39 (16.7)	32 (21.2)	110 (34.0)	64 (27.6)	36 (23.4)
36—less 59	152 (76.4)	106 (99.1)	194 (83.3)	119 (78.8)	214 (66.0)	168 (72.4)	118 (76.6)
Sex							
Male	97 (48.7)	58 (54.2)	123 (52.8)	82 (54.3)	156 (49.4)	110 (48.2)	72 (42.9)
Female	102 (51.3)	49 (45.8)	110 (47.2)	69 (45.7)	160 (50.6)	118 (51.8)	80 (57.1)
Anaemia status							
Normal	76 (38.4)	22 (20.6)	82 (30.7)	21 (13.9)	81 (25.2)	18 (7.8)	37 (24.2)
Anaemia	122 (61.6)	85 (79.4)	150 (69.3)	130 (86.1)	241 (74.8)	212 (92.2)	116 (75.8)
Weight for age (WAZ)							
Normal	168 (85.3)	86 (80.4)	169 (73.5)	122 (82.4)	252 (81.6)	188 (84.7)	114 (77.6)
Underweight	29 (14.7)	21 (19.6)	61 (26.5)	26 (17.6)	57 (18.4)	34 (15.3)	33 (22.4)
Malaria infection							
No	104 (52.3)	43 (40.2)	121 (51.9)	118 (78.1)	137 (42.3)	188 (81.0)	116 (75.3)
Yes	95 (47.7)	64 (59.8)	112 (48.1)	33 (21.9)	187 (57.7)	44 (19.0)	38 (24.7)
Fever							
No	156 (78.4)	83 (77.6)	168 (72.1)	105 (69.5)	264 (81.5)	185 (79.7)	104 (67.5
Yes	43 (21.6)	24 (22.4)	65 (27.9)	46 (30.5)	60 (18.5)	47 (20.3))	50 (32.5)
Malaria cases							
Asymptomatic	168 (84.4)	91 (85.0)	194(83.3)	138 (91.4)	280 (86.4)	216 (93.1)	133 (86.4)
Clinical malaria	31 (15.6)	16 (15.0)	39 (16.7)	13 (8 .6)	44 (13.6)	16 (6.9)	21 (13.6)

Using the PCD data, the unadjusted comparison of no null percentage malaria episode numbers (0, 1 or 2 +) by age groups, gender, anaemia, and weight-for-age status for each transmission period indicated a significant difference in gender in 2013 (February to June), in WAZ status in 2014 (June to October) and in anaemia status in 2015 (June to November) (all p < 0.05). There was no significant difference elsewhere (Table 2).

**Table 2:** Comparison of numbers of cases for malaria multiples episodes by risk factors and by follow-up periods for children under 5 years from 2013 to 2017 in Dangassa

Follow-up periods	Episodes numbers	Age groups in months			Gender			Anaemia Status			WAZ		
		6–35	36–59	*p-*value	Male	Female	*P-*value	Normal	Anaemia	*p-value*	Normal	Under-weight	*p-*value
		nb. Cases	nb. Cases		nb. Cases	nb. Cases		nb. Cases	nb. Cases		nb. Cases	nb. Cases	
Feb–Jun 2013	0	67	68	0.58	64	71	0.04*	88	46	0.76	119	15	0.09
	1	14	15		12	17		20	9		23	6	
	2 +	3	1		4	0		2	2		2	2	
Jun–Oct 2013	0	34	45	0.18	34	45	0.40	52	26	0.93	69	9	0.73
	1	38	27		32	33		43	22		55	10	
	2 +	12	12		14	10		15	9		20	4	
Oct 2013–Jun 2014	0	45	56	0.19	46	55	0.76	65	35	0.40	89	11	0.27
	1	29	19		25	23		30	18		38	10	
	2 +	10	9		9	10		15	4		17	2	
Jun–Oct 2014	0	17	45	0.11	32	30	0.63	47	15	0.62	43	19	0.04*
	1	11	26		22	15		29	8		33	4	
	2 +	11	10		10	11		18	3		14	7	
Oct 2014–Jun 2015	0	44	29	0.00*	39	34	0.33	37	34	0.21	48	22	0.30
	1	0	18		11	7		12	6		15	3	
	2 +	0	7		2	5		2	5		6	1	
Jun–Nov 2015	0	93	75	0.00*	79	85	0.12	138	30	0.03*	133	28	0.51
	1	0	23		16	7		22	1		21	2	
	2 +	0	10		4	6		10	0		8	2	
Nov 2015–Jun 2016	0	108	101	0.00*	99	106	0.10	157	50	0.43	156	43	0.15
	1	0	5		3	2		4	1		5	0	
	2 +	0	3		3	0		3	0		3	0	
Jun–Dec 2016	0	103	99	0.00*	93	105	0.17	184	17	0.62	164	30	0.38
	1	0	9		6	3		8	1		7	2	
	2 +	0	5		4	1		5	0		5	0	
Dec 2016–Mar 2017	0	59	74	–	61	70		104	28		100	28	-

*nb* Cases: Cases number; * Statistical significance

The peak of incidence risk (IR) for one (IR = 95.73 in 1000 person-months, n = 65) and 2 + (IR = 35.35 in 1000 person-months, n = 24) episodes was observed in 2013 during the high transmission period (June to October). IR decreased to zero case at the end of the follow-up in March 2017. The lowest IR for 1 episode and 2 + episodes were observed in the second half time of the study follow-up period in 2016 (June–December) with 9 cases (IR = 6.78 in 1000 person-months) and 5 cases (IR = 3.77 in 1000 persons-months), respectively (Table 3).

**Table 3:** Incidence rate in 1000 person months of malaria episodes numbers for children under 5 years from 2013 to 2017 in Dangassa

Follow-up periods	Episodes numbers					
	1			2 +		
	nb Cases	nb. person months	Incidence rate	nb Cases	nb. person months	Incidence rate
Feb–Jun 2013	29	521	55.66	4	521	7.68
Jun–Oct 2013	65	679	95.73	24	679	35.35
Oct13–Jun 2014	48	916	52.40	19	916	20.74
Jun–Oct 2014	37	706	52.41	21	706	29.75
Oct14–Jun 2015	18	547	32.91	7	547	12.80
Jun–Nov 2015	23	923	24.92	10	923	10.83
Nov15–Jun 2016	5	1058	4.73	3	1058	2.84
Jun–Dec 2016	9	1327	6.78	5	1327	3.77
Dec16–Mar 2017	0	389	0.00	0	389	0.00

*nb* Cases: Cases number, *nb* person Number Person Months

**Fig. 3:**
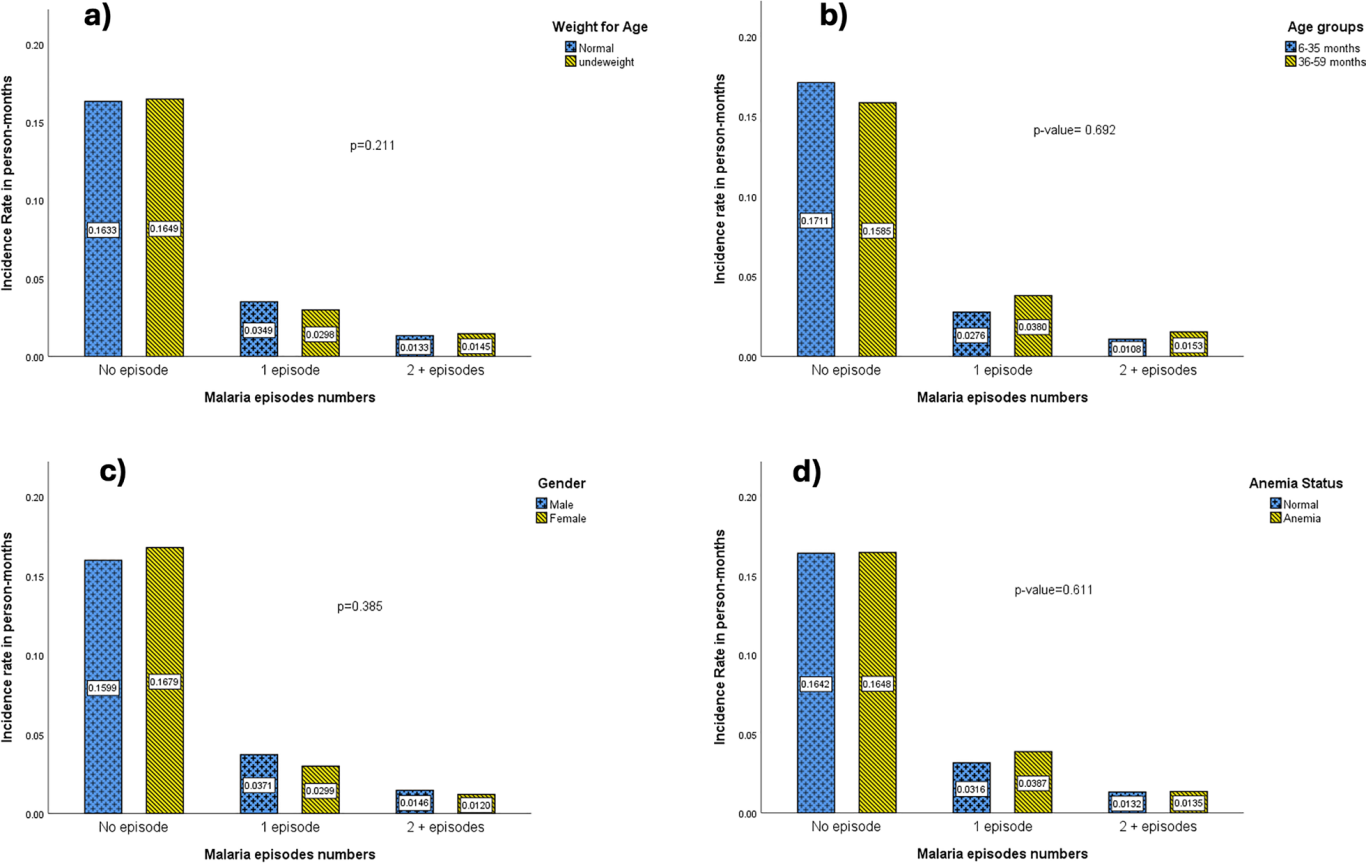
Univariate comparison for having higher episode numbers by **a** Age, **b** Gender, **c** Anaemia and **d** Weight for Age groups percentage within each malaria episodes numbers during the follow-up’s time in person year

The univariate analysis of risk factors between age groups indicated that children 36–59 months were more likely to have higher numbers of malaria episodes than 6–35 months old. Males were more likely to experience a higher number of multiple malaria episodes as well as anaemia compared to females. However, none of the differences was significant (all p > 0.05) (Fig. 3a–d).

After adjusting for all other parameters, the odds ratio (OR) for having a higher number of malaria episodes during the high transmission period was higher than in the low transmission period [OR = 3.23, 95% CI (2.45–4.26), p = 0.000]. The children with anaemia were more likely to have higher numbers of malaria episodes [OR = 1.6, 95% CI (1.12–2.30), p = 0.011] than those with normal haemoglobin levels (Table 4).

**Table 4:** Univariate and Multivariate Odds Ratio by risk factors of having higher number of malaria episodes in person years using Repeated Ordinal logistics Regression model (Repeated Proportional odds Model) with time of exposure as the offset

	GEE: repeated proportional odds model					
	Univariate (unadjusted)			Multivariate (adjusted)		
	OR	95%CI OR	*p-*value	OR	95%CI OR	*p-*value
Age groups (months)						
6–35 (ref)	1	1	0.692	1	1	0.890
36–59	0.91	0.58–1.43		0.97	0.64–1.47	
Gender						
Male (ref)	1	1	0.385	1	1	0.336
Female	1.31	0.71–2.43		1.29	0.78–2.15	
Anaemia status						
Normal (ref)	1	1	0.611	1	1	0.011
Anaemia	1.13	0.71–1.78		1.60	1.12–2.30	
Weight for age (WAZ)						
Normal (ref)	1	1	0.211	1	1	0.084
Underweight	1.38	0.84–2.26		1.46	0.95–2.23	
Transmission period						
Low (ref)	1	1	0.0001*	1	1	0.0001*
High	3.01	2.37–3.84		3.23	2.45–4.26	

*OR* odd ratio**, ***CI* Confidence intervals, * Statistically significant difference

**Fig. 4:**
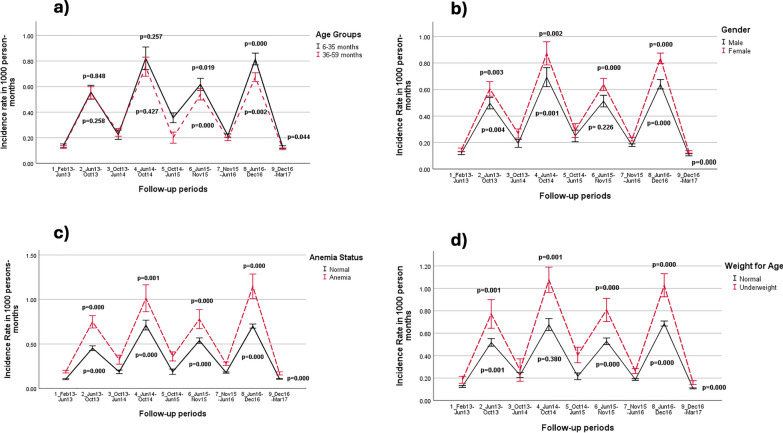
Trend of the predicted Incidence Rate in person-months of children under 5 years for Malaria 2 + episodes by **a** Age, **b** Gender, **c** Anaemia and **d** Weight for Age groups in Dangassa from 2013 to 2017

During low and high transmission periods, the trends of the predicted IR in person-months among the categories of each risk factor are shown in Fig. 4a–d. The trend indicated a significant difference between gender, WAZ, and anaemia status during the high transmission period. However, during the low transmission period, anaemic and underweight children were more likely to have 2 + malaria episodes (all p < = 0.001) except for underweight children in the follow-up period from October 2013 to June 2014, (p = 0.38).

In contrast, the relative risk of two or more episodes of anaemia in low transmission periods (RR = 1.67, 95%CI [1.15–2.42], p = 0.007) was slightly higher than in high transmission periods (RR = 1.58, 95%CI [1.09–2.29], p = 0.016) (Table 5).

**Table 5:** Predicted Incidence Rate in 1000-person-months and Relative Risk by transmission periods (low and High) and by risk factors of Malaria multiples episodes (2 + episodes) among children under 5 years from 2013 to 2017 in Dangassa

	Low transmission				High transmission			
	IR	RR	CI 95%	*p-*value	IR	RR	CI 95%	*p-*value
Age group (months)								
36–59	0.18	0.90	0.60–1.37	0.616	0.69	1.09	0.72–1.65	0.682
6–35 (ref)	0.20				0.63			
Gender								
Female	0.21	1.23	0.74–2.05	0.426	0.73	1.25	0.75–2.08	0.391
Male (ref)	0.17				0.58			
Anaemia status								
Anaemia–yes	0.27	1.8	1.24–2.61	0.002*	0.86	1.41	0.97–2.05	0.071
Anaemia-No (ref)	0.15				0.61			
Weight for age (WAZ)								
Underweight	0.26	1.53	0.99–2.35	0.053	0.32	1.52	0.99–2.34	0.057
Normal (ref)	0.17				0.21			

*IR* Incidence rate, *RR* Relative risk, * Statistically significant difference

## Discussion

In endemic countries, malaria is a major public health problem, particularly in low-income countries [16]. Despite the complexity of the interaction between malaria, anaemia, and nutritional status [1], understanding their relationship is vital for the development of strategies that will reduce child morbidity and mortality. Using repeated cross-sectional surveys and passive case detection at a community health centre for five successive years, the relationship between anaemia, underweight, and multiple malaria episodes among children under the age of five in Dangassa was assessed.

Based on the baseline descriptive analysis, symptomatic malaria cases varied between CSSs and years. The number of cases was higher at the end of the rainy season than at the beginning. In malaria-endemic areas, the parasite density is usually higher at the end than the start of the rainy season [17]. During this period, the entomological inoculation rate is particularly high [18]. Toure et al*.* in Sélingué, Mali [19] showed that malaria is seasonal in Mali, with a peak at the end of the rainy season. Although the survey was conducted during the dry season, the prevalence of symptomatic malaria was compared to the prevalence at the beginning of the transmission season. As Dangassa is a riverine village where transmission is particularly prolonged (5 to 6 months), high spatial and temporal clustering of *P. falciparum* infection has been demonstrated during the dry season [20].

The significant difference between the percentage of malaria episodes numbers by anaemia status in June to November 2015 indicated a high percentage among no anaemia children. However, anaemia is frequently associated with clinical malaria particularly the severe form in area where the disease is endemic [21]. The introduction of both seasonal malaria chemoprevention (SMC) and LLINs universal coverage in Dangassa for children less than 5 years could explained this change in the risk of clinical malaria with respect to anaemia status [22]. Indeed, studies have shown a positive impact of SMC on the reduction of malaria transmission and malaria indicators such as anaemia in children in West Sahelian Africa [23, 24].

The peak of malaria incidence was observed in 2013 during the period of high transmission (June to October), with respectively 65 children ((IR = 95.73 in 1000 person-months) for multiple episodes and 24 cases (IR = 35.35 in 1000 person-months) for those with 1 episode. Most malaria cases detected by PCD in Dangassa are symptomatic and occur during the five- to six-month transmission season (June to November) [25]. Also, Ateba et al*.* reported a higher number of cases during the period when SMC was not distributed compared with the period when SMC was implemented in the same area [26].

However, the frequency of malaria episodes decreased significantly between 2013 and 2017 within the cohort. As a result, (i) the intensification of malaria control strategies in Dangassa, including the distribution of LLINs and the introduction of SMC, have significantly reduced the burden of malaria in Dangassa [27]; (ii) Community sensitization and participation in the study; (iii) Qualified staff deployed within the framework of the International Centre of Excellence for Malaria Research in West Africa (ICEMR-1) project; (iv**)** Health centre use by residents.

According to these results, underweight is associated with a higher number of episodes compared to normal children 10% error [OR = 1.46, 95% CI (0.95–2.23), p = 0.084]. Almeida et al*.* [28], in a rural community in the Amazon region, demonstrated that malaria-induced anorexia and vomiting during the acute phase of the disease, associated with insufficient micronutrients, in children under 5 years of age in a specific endemic context, could lead to a delay in the child's physical development after several episodes of malaria.

The adjusted model showed a significant association between multiple episodes in anaemic and normal children [OR = 1.6, 95%CI (1.12–2.30), p = 0.011]. Thus, in high transmission areas such as Dangassa, where the disease is prevalent, many young children are anaemic, and people infected with the disease may receive a single bite each day, exposing them to repeated episodes of the disease. Consequently, in these contexts, severe malarial anaemia (haemoglobin < 7 g/dl) [29], in young children, a blood transfusion is required, which characterizes a compromised rapid recovery of the anaemia, since malaria infection causes haemolysis of parasitized and non-parasitized erythrocytes, and dyserythropoiesis of the bone marrow [30].

The occurrence of multiple episodes was significantly associated with anaemic and underweight children during the low transmission period. Anaemia has a complex aetiology, including single- and multifactorial causality [31]. Thus, the high occurrence of malaria in Dangassa during the low transmission period in anaemic and underweight children is associated probably through protein-energy and vitamin deficiencies. In 2018, Aimée et al., from the Democratic Republic of Congo, reported that iron deficiency can negatively affect children's weight growth [32]. Mariken et al*.*, measuring the association between nutritional status and malaria incidence in young children before the malaria transmission season, found that, underweight was associated with a higher incidence of clinical malaria in Burkina Faso [33].

The risk of having multiple malaria episodes during the high transmission period was 3.23 times higher than during the period of low transmission [CI (2.45–4.26); p = 0.000]. Although the number of multiple malaria episodes increase following exposure to malaria transmission, the rainfall, which is low during periods of low transmission and high during periods of high transmission, is an important factor in the dynamics of malaria transmission. Toure et al*.* [19] reported the same results and showed a clear seasonal pattern, with a reduced below 50% malaria incidence during the dry season.

The study has the limitation that, because the data were analysed secondary, some anthropometric parameters needed, such as height and the upper arm circumference to determine children’s nutritional status, were not collected. Consequently, some variables related to these indices could not be incorporated into these analysis.

## Conclusion

The presence of multiple malaria episodes was significantly associated with nutritional status (anaemia and low weight-for-age) in children during both high and low transmission periods. However, this relationship was more pronounced during the dry season (“low transmission” period). A further study including more parameters are necessary to support this hypothesis.

## Data Availability

The datasets generated during and/or analysed during the current study are available from the corresponding author on reasonable request.

## Abbreviations

CI: Confident interval
CSSs: Cross sectional surveys
GEE: Generalized estimating equation
Hb: Haemoglobin
HTP: High transmission period
ICEMR-WA: International Centre of Excellence for Malaria Research in West Africa
IR: Incidence rate
LLINs: Insecticide-impregnated mosquito nets
LTP: Low transmission period
MIS: Malaria Indicator Survey
NMCP: National Malaria Control Program
OR: Odd ratio
PCD: Passive case detection
RDT: Rapid diagnostic test
RR: Relative risk
SD: Standard deviation
SMC: Seasonal Malaria Chemoprevention
WAZ: Weight-for-age
WHO: World Health Organization
